# Melatonin action in *Plasmodium* infection: Searching for molecules that modulate the asexual cycle as a strategy to impair the parasite cycle

**DOI:** 10.1111/jpi.12700

**Published:** 2020-10-28

**Authors:** Pedro H. S. Pereira, Celia R. S. Garcia

**Affiliations:** ^1^ Department of Clinical and Toxicological Analyses School of Pharmaceutical Sciences University of São Paulo São Paulo Brazil

**Keywords:** antimalarials, melatonin, *Plasmodium*, synchronicity

## Abstract

Half of the world's population lives in countries at risk of malaria infection, which results in approximately 450,000 deaths annually. Malaria parasites infect erythrocytes in a coordinated manner, with cycle durations in multiples of 24 hours, which reflects a behavior consistent with the host's circadian cycle. Interference in cycle coordination can help the immune system to naturally fight infection. Consequently, there is a search for new drugs that interfere with the cycle duration for combined treatment with conventional antimalarials. Melatonin appears to be a key host hormone responsible for regulating circadian behavior in the parasite cycle. In addition to host factors, there are still unknown factors intrinsic to the parasite that control the cycle duration. In this review, we present a series of reports of indole compounds and melatonin derivatives with antimalarial activity that were tested on several species of *Plasmodium* to evaluate the cytotoxicity to parasites and human cells, in addition to the ability to interfere with the development of the erythrocytic cycle. Most of the reported compounds had an IC50 value in the low micromolar range, without any toxicity to human cells. Triptosil, an indole derivative of melatonin, was able to inhibit the effect of melatonin in vitro without causing changes to the parasitemia. The wide variety of tested compounds indicates that it is possible to develop a compound capable of safely eliminating parasites from the host and interfering with the life cycle, which is promising for the development of new combined therapies against malaria.

## 1 *PLASMODIUM FALCIPARUM* LIFECYCLE AND DRUG RESISTANCE

**Figure 1:**
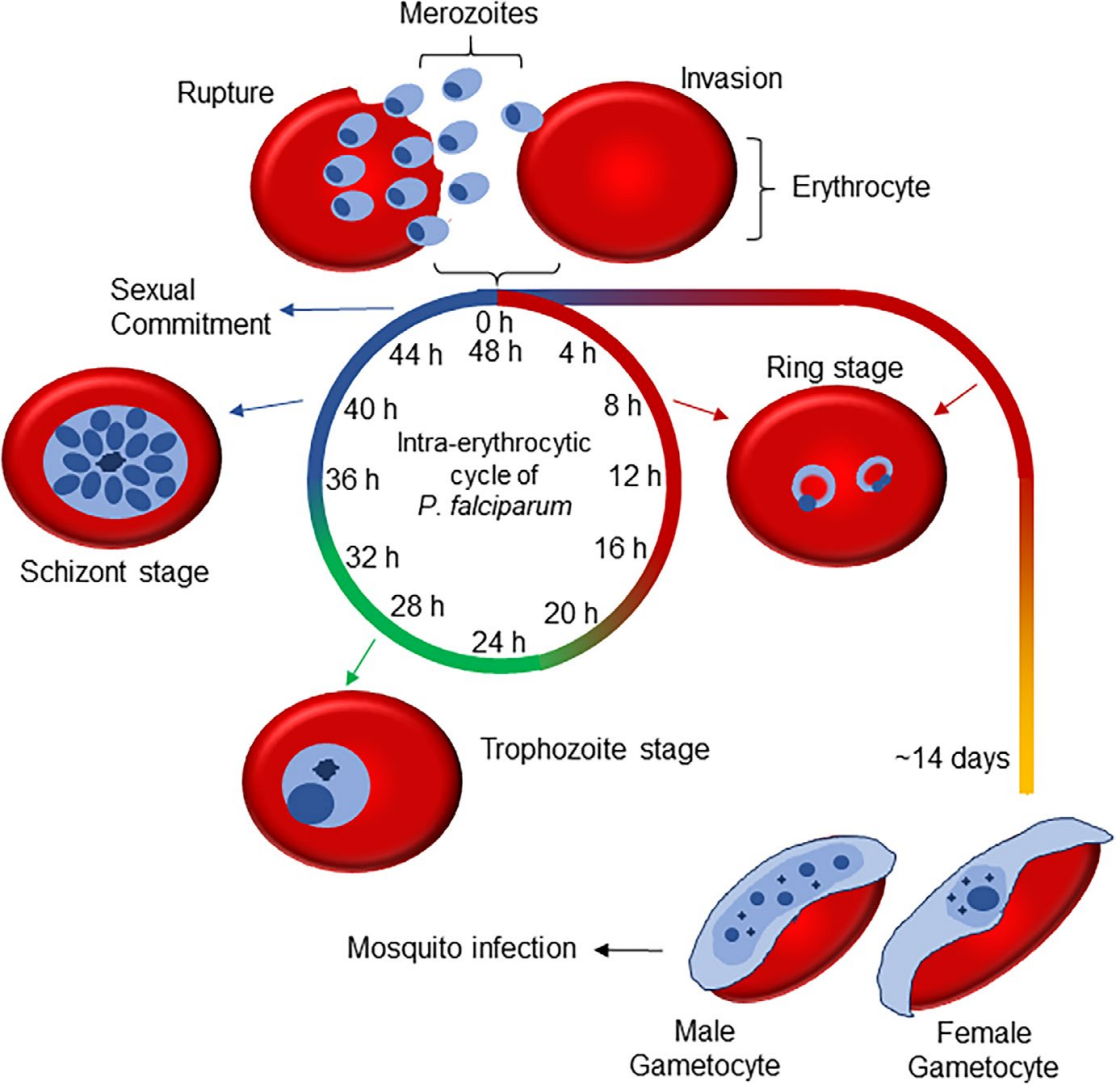
*Plasmodium falciparum* intraerythrocytic cell cycle. The life cycle of *Plasmodium falciparum* begins with the bite of a female *Anopheles* mosquito, which injects a small amount of saliva containing parasites into the bloodstream. At this stage, the parasite is called a sporozoite, and they migrate through the bloodstream to the liver where they invade hepatocytes. Sporozoites within hepatocytes develop until they differentiate into schizonts. Schizonts divide by asexual reproduction, generating merozoites, which are the phase responsible for erythrocyte invasion. After invasion, the merozoites differentiate into rings (or young trophozoites). The rings gradually increase in cell volume and metabolism, until they become mature trophozoites. At this stage, there is an increase in DNA replication and preparation for schizogony (asexual cell division). The multinucleate stage is called a schizont, and it develops until the segmentation of the multiple nuclei and the formation of new merozoites occurs. The rupture of schizonts releases more merozoites into the bloodstream, which invade new erythrocytes and continue the erythrocytic cycle. A small percentage of merozoites at schizont stage are committed to differentiate into gametocytes after parasite invasion to RBCs. The factors that determine which, when and how many merozoites participate in this process are not well established to date. Gametocytes develop within erythrocytes for approximately 15 days and through 5 different stages until they are captured by a female *Anopheles* mosquito during feeding

Malaria is caused by a protozoan of the genus *Plasmodium,* and the species *P falciparum* is responsible for the most serious human parasitic disease. It is estimated that 3.3 billion people live in areas at risk of infection and 216 million cases were reported in 2017, leading to more than 450,000 deaths.^1^ The asexual cycle of *P falciparum* occurs in the human host, with infection initiated by the bite of the female *Anopheles* mosquito, which injects sporozoites together with saliva in the host.^2^ Once in the bloodstream, sporozoites migrate to the liver and invade hepatocytes, where they can remain inactive or replicate asexually, forming a large number of merozoites in the host cell.^3^, ^4^ The release of merozoites into circulation marks the beginning of the erythrocytic stages (Figure 1). Merozoites invade erythrocytes and develop within the parasitophorous vacuole, undergoing various biochemical and morphological transformations, which can be identified by three stages referred to as ring, trophozoite and schizont.^5^ Young trophozoites, also called “rings” because of their morphology, are small and have low metabolic activity until they develop into fully grown trophozoites. In the trophozoite stage, metabolic activity is high and there is an increase in cell volume and hemoglobin digestion in preparation for schizogony. In the schizont stage, the parasite undergoes multiple DNA replication steps and successive mitotic divisions. At this point, newly formed merozoites wait to exit the host cell simultaneously. Mature forms of the erythrocyte stages adhere to endothelial cells to avoid contact with the immune system in the spleen.^6^ The rupture of erythrocyte releases all internal contents, including new merozoites, into the bloodstream, allowing for reinvasion of red blood cells and a continuation of the intraerythrocytic cycle. The simultaneous rupture of erythrocytes causes an intense peak in the immune response every 24 or 48 hours, which is a clinical feature of *Plasmodium* infection with symptoms including high fever and chills at well‐defined intervals that coincide with the duration of the intraerythrocytic cycle.^7^, ^8^ Protozoan erythrocyte invasion, their development within the cell, and erythrocyte rupture occur in a coordinated manner, such that all parasites complete the cycle at a well‐defined time of day.^9^ The most accepted hypothesis for this phenomenon is that the simultaneous release of parasites into the bloodstream can provide protection against the host immune system.^9^, ^10^, ^11^

During reinfection of erythrocytes, a small percentage of merozoites differentiate into gametocytes, the infective stage of the mosquito vector.^12^ The mechanism that determines which parasites will undergo this process is still unclear, but it is known that some host cues play an essential role in this differentiation process. It takes 10‐12 days and five morphological stages of maturation until a gametocyte‐committed merozoite can invade an erythrocyte and differentiate into a single mature male or female gametocyte, which remains circulating in peripheral blood vessels for several days. During mosquito feeding, gametocytes are ingested and complete their maturation into gametes inside the mosquito intestine, after which fertilization and zygote formation occur.^13^ The zygote migrates to the intestinal epithelium, where it develops into an oocyst. The rupture of the oocyst releases sporozoites, which migrate to the salivary gland and are injected into the human bloodstream during mosquito feeding, completing the cycle.^14^

For several decades, the fight to control malaria has been challenged by the emergence of parasites that are resistant to antimalarials used on a large scale. First, the appearance of chloroquine resistance compromised this effective treatment, which was followed successively by resistance to sulfadoxine + pyrimethamine, mefloquine, and, more recently, artemisinins. Mefloquine + artesunate‐based therapy, one of the first artemisinin derivatives, began being replaced in 2007 after an increasing number of treatment failures; it was substituted by the combination of piperaquine (PPQ) + dihydroartemisinin (DHA).^15^, ^16^ The first warning signs for PPQ + DHA came in 2009, when reports that patients were taking longer than expected to respond to treatment began, which culminated in the failure of first‐line treatment with PPQ + DHA in recent years.^17^

One of the main reasons for the recent reduction in the number of malaria cases is the excellent clinical efficacy of combined artemisinin therapies (ACTs), which have been adopted worldwide as a first‐line treatment, together with the growing number of mosquito vector control measures.^1^ However, treatment with ACTs faces an obstacle due to the increasing number of artemisinin‐resistant parasites that have emerged and spread throughout South‐East Asia. ACTs, in turn, led to the failure of DHA and PPQ in Cambodia, where parasites are now resistant to both drugs.^18^, ^19^ Therefore, there is an urgent need to identify alternative drugs that can be used in mass treatment campaigns to reduce the local burden of malaria and block the spread of resistant strains. The main objective is to eliminate malaria from this region completely before resistance spreads to Africa, where it would have devastating consequences for public health.^20^, ^21^

## 2 MELATONIN ACTION IN *PLASMODIUM FALCIPARUM* INFECTION: SEARCHING FOR MOLECULES THAT MODULATE THE ASEXUAL CYCLE AS A STRATEGY TO IMPAIR THE PARASITE CYCLE

The in vivo maturation process of the parasite occurs in an extremely coordinated way, in which the schizonts rupture, releasing merozoites that invade new erythrocytes in a highly synchronized manner at intervals of 24 hours, depending on the *Plasmodium* species.^11^ This means that for rupture and invasion events to occur simultaneously in different cells, the parasites must develop through the intraerythrocytic stages in a coordinated manner so that at the end of the cycle, the vast majority of schizonts are ready to rupture at the same time. The host hormone melatonin can modulate the synchrony of parasite development in the rodent *Plasmodium chabaudi* and the human parasite *Plasmodium falciparum*. Of note, pinealectomized mice infected by *P chabaudi* lose the ability to synchronize. Interestingly, the nonsynchronous rodent malaria parasites are not affected by melatonin, as was previously reported in studies using *P yoelli* and *P berghei* as models.^22^

**Figure 2:**
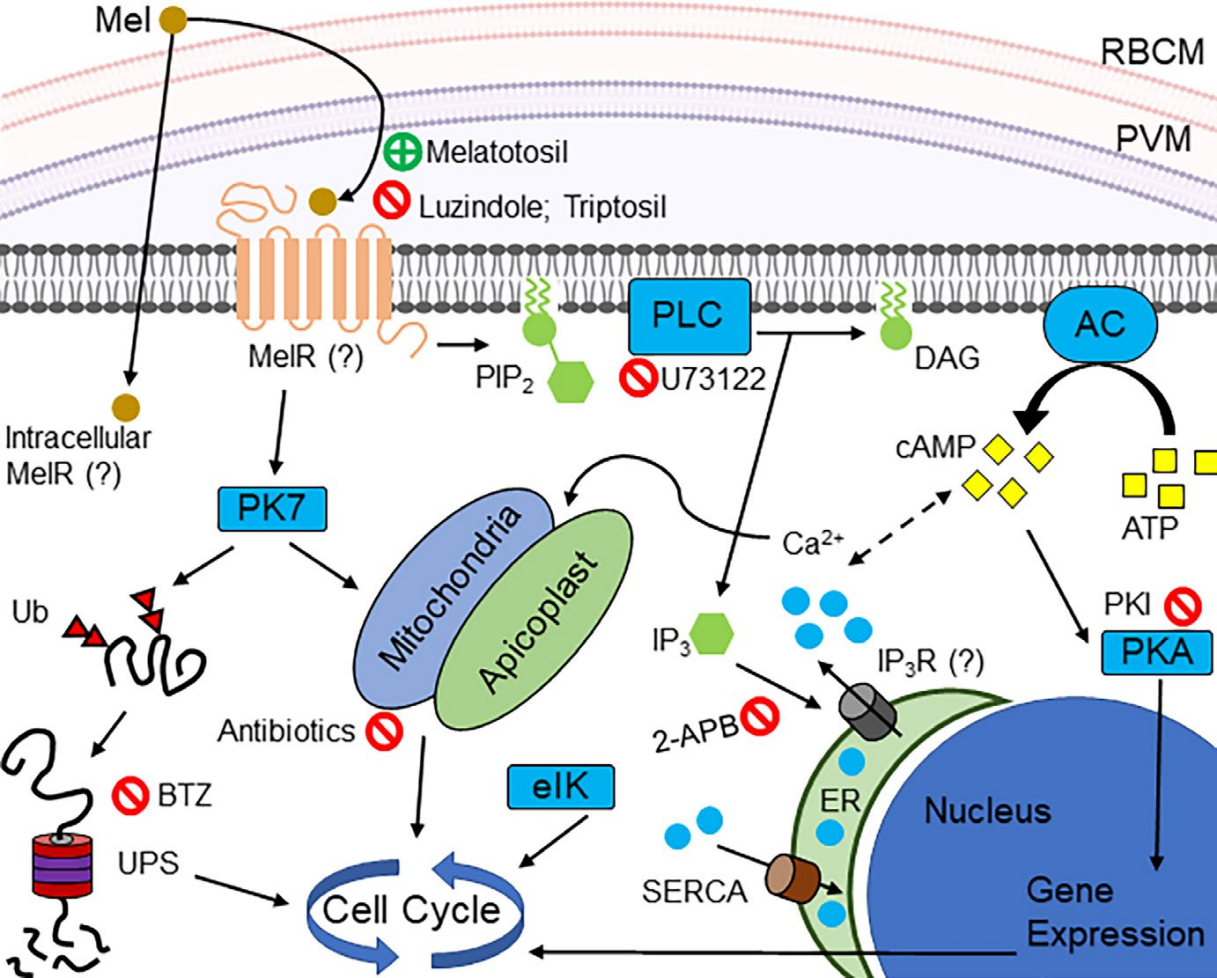
Melatonin signaling pathway in *Plasmodium falciparum*. Melatonin binds to an unknown receptor on the parasite membrane. Signaling depends on the action of a PLC that produces IP_3_. IP_3_ binds to another unknown receptor in the endoplasmic reticulum and causes the release of Ca^2+^ from the organelle into the cytoplasm. Ca^2+^ activates a series of effector proteins, such as proteases and kinases, that control gene expression and cell cycle progression. PK7 plays an essential role, regulating gene expression and the ubiquitin proteasome system. Steps that can be pharmacologically inhibited with the effect of altering the parasite cell cycle are highlighted in red. 2‐APB: 2‐aminoethyl diphenylborinate; AC: Adenylate cyclase; ATP: Adenosine triphosphate; BTZ: bortezomib; cAMP: cyclic adenosine monophosphate; DAG: diacylglycerol; eIK: eukaryotic initiation factor; ER: endoplasmic reticulum; IP_3_: inositol 1,4,5‐triphosphate; IP3R: IP_3_ receptor; Mel: melatonin; MelR: melatonin receptor; PIP2: phosphatidylinositol 4,5‐bisphosphate; PK7: protein kinase 7; PKA: protein kinase A; PKI: protein kinase A inhibitor; PLC: phospholipase C; PVM: parasitophorous vacuole membrane; RBCM: red blood cell membrane; SERCA: sarco/endoplasmic reticulum Ca^2+‐^ATPase; Ub: ubiquitin; UPS: ubiquitin proteasome system

*Plasmodium falciparum* protein kinase 7 (PfPK7) is an orphan kinase with no orthologs in mammalian cells. The C‐terminal PfPK7 region displays similarity to the protein kinase activated by mitogen kinase (MAPKK), while the N‐terminal region has similarity to the fungal protein kinase A.^23^ The pfpk7 gene knockout has a slow parasite growth phenotype that is caused by a reduction in the number of merozoites in each cycle.^24^ The exposure of the PfPK7 knockout strain (PfPK7‐) to melatonin does not alter the proportion of asexual stages.^25^ Recently, a comparative study of the PfPK7‐ and Pf3D7 strain phosphoproteomes was carried out in schizonts, resulting in the identification of 3,875 phosphorylation sites in 1,047 proteins..^26^ In addition to PfPK7, the *P falciparum* eukaryotic translation initiation factor kinase (Pfeik1) has been recently identified as another central kinase that transduces melatonin signaling in melatonin‐induced parasite synchronization.^27^ The roles and potential cross‐activation of the Pfeik1 and PfPK7 kinases in parasite synchronization remain interesting questions to be investigated. Of interest, the activation of protein kinase A (PKA) in downstream melatonin signaling pathways occurs in both *P falciparum* ^28^ and *P chabaudi*.^29^ Decoding how the above kinases interact to culminate in parasite cell cycle progression is an interesting open question that could lead to new ways to block the malaria cycle (Figure 2).

Employing transcriptomic methodology such as RNA‐Seq, Lima et al (2016) reported that melatonin modulates the expression of 38 genes in the 3D7 wild‐type parasite strain compared to PfPK7‐knockout parasites.^30^ The genes in the ubiquitin proteasome system (UPS) were among those with altered expression.^31^ Relatedly, the PfNFYB transcription factor is one of the targets of melatonin‐induced ubiquitination in *P falciparum*.^32^, ^33^ In addition, the action of melatonin on the mitochondrial dynamics of *P falciparum* has also been reported. Melatonin is able to modulate the expression of three genes (two GTPase dynamins, PfDYN1 and PfDYN2, and the mitochondrial fission protein PfFIS1) possibly related to mitochondrial fission in a stage‐specific manner.^34^ Melatonin can increase the expression of PfDYN1 and PfFIS1, which could suggest that accelerated cell cycle progression occurs via the regulation of mitochondrial distribution to new cells.

The mechanism of melatonin signal transduction in *P falciparum* involves the activation of phospholipase C, which promotes the generation of inositol triphosphate (IP_3_), leading to an increase in the cytosolic Ca^2+^.^35^ The known IP_3_ receptor sequences are widely used in bioinformatics techniques as probes to search for uncharacterized receptors. However, all attempts to identify these receptors in apicomplexan parasites have failed and a canonical candidate for the *Plasmodium* IP_3_ receptor has not been identified.^36^

Temporarily controlled administration of melatonin can be used as a form of treatment for physiological disorders.^37^, ^38^, ^39^, ^40^, ^41^ Melatonin can act as an intracellular antioxidant, participating in the processes of aging and the formation of free radicals, and its high concentration in the mitochondria of eukaryotic cells supports these roles.^42^, ^43^, ^44^, ^45^ In fact, mitochondrial concentrations of melatonin are not affected by extracellular concentrations, and there are reports that purple bacteria, which gave rise to the mitochondria of modern eukaryotes, are capable of producing this hormone.^46^, ^47^

Recently, the periodicity of two *Plasmodium* species cycles and their relationship with the host's circadian clock were analyzed, aiming to separate parasite‐intrinsic aspects from the host‐specific aspects contributing to cell cycle regulation. Filipa Rijo‐Ferreira et al (2020) identified a relationship between the *Plasmodium chabaudi* circadian clock and the transcriptional regulation of a group of 174 genes that peak every 18 hours, indicating a parasite‐intrinsic mechanism of cell cycle regulation.^48^ However, this periodicity does not explain synchronization in a population, only in isolated parasites. Similarly, Lauren M. Smith et al (2020) identified the harmonic expression of a set of genes in *P falciparum* that was equivalent to half the duration of the cell cycle and resulted in two peaks of expression. In addition, the results were consistent with strains that have different total intraerythrocyte cycle durations.^49^ Both of these studies showed that there is an intrinsic biological clock that renders individual parasites capable of regulating the duration of the erythrocytic cycle; however, at the population level, aspects of the host may be necessary. One of the genes that have a circadian expression pattern, serpentine receptor 10 (SR10), is a promising candidate as a key to link aspects of the host with the intrinsic clock of the parasites; knockout of this gene shortens the *P chabaudi* cycle to 2‐3 hours and various cellular processes are deregulated, such as DNA replication and the ubiquitin proteasome system.^50^

## 3 MELATONIN AND ITS DERIVATIVES AS ANTIMALARIALS

Melatonin and derivatives of melatonin have been shown to affect malarial infection. In vivo experiments using luzindole in combination with suboptimal doses of chloroquine have shown promise; treatment of mice with luzindole and chloroquine has shown a clear synergistic effect, in which 25% of the animals remained alive on day 10 after treatment with chloroquine or luzindole individually, while 75% remained alive when the two drugs were administered together. There was also a decrease in parasitemia when the two drugs were used together compared to each drug separately, and as luzindole is not toxic to the parasite, the synergistic effect can be attributed to their ability to interfere with the synchronization of the erythrocytic cycle and improve the effectiveness of the host immune system.^22^

It is important to note that the indole precursors of melatonin, such as serotonin, N‐acetyl serotonin, and tryptamine, also affect the development of the intraerythrocytic cycle in a similar way. By contrast, IAA (indole 3‐acetic acid), another indole compound that regulates several physiological processes in plants, is not able to interfere in the erythrocytic cycle. While melatonin and N‐acetyl serotonin increase the expression of UPS genes, IAA does not, and instead, it decreases the expression of cullin (PF08_0094), another gene in that system. These data indicate that there is a degree of specificity in this class of compounds as regulators of different functions in the cell cycle of *P falciparum*.^51^

Recently, Dias, B. K. M. et al (2020) reported the potential of ten new synthetic indole derivatives as antimalarials by investigating their ability to act as agonists or antagonists of a putative melatonin receptor in *P falciparum*. While growth inhibition occurred at low micromolar levels, the compound melatotosil was identified as a potential agonist of a putative melatonin receptor in *P falciparum*. The characteristic effects of increased *P falciparum* parasitemia after treatment with melatonin were evident after treatment with melatotosil. In addition, coincubation of melatonin and melatotosil inhibited the expected effect, which indicates competition between the two molecules for the same target. By contrast, the compound triptosil had no effect on parasitemia but was able to abolish the effect of melatonin.^27^

Teguh et al (2013) characterized a series of conjugated quinoline and indole compounds that had antimalarial activity, demonstrating a mechanism of action that differed from those observed with quinolines, such as chloroquine, which likely affected mitochondrial functions via changes in the membrane potential of these organelles.^52^

Fernandez et al (2009) reported on indole alkaloids extracted from *Fliersia* sp. using HPLC and tested the antimalarial activity of these compounds against *P falciparum* strains resistant to chloroquine and in cytotoxicity assays using HEK293 cells. The results indicated that three compounds were capable of inhibiting parasite growth by 50% at concentrations between 80 nM and 142 nM, and one compound, dimethylisoborreverine, had a potency comparable to artemisinin, with an IC50 of 20 nM.^53^

A series of compounds derived from triazine‐indole were synthesized by Kgokong et al (2005) and tested for antimalarial capacity. The group concluded that the addition of a trifluoromethyl group was responsible for the IC50 of 30 nM in both resistant and chloroquine‐sensitive strains, a value that is similar to that of mefloquine. Removal of the trifluoromethyl group causes the compound to have no antimalarial activity, raising the IC50 value to 0.4 mM.^54^

Agarwal et al (2005) synthesized 24 indole compounds and tested the ability of each to inhibit parasite maturation. Six compounds containing N‐methyl piperazine have been described as ring substitutes with good antimalarial activity, capable of inhibiting ring maturation in schizonts. The compounds were 10 times more effective than pyrimethamine in preventing the erythrocyte cycle from maturing.^55^

Another set of indole derivatives was tested by Schuck et al (2014), who investigated their toxicity and ability to interfere with the intraerythrocytic cycle of *P falciparum* in vitro in a similar role to that of melatonin. Two compounds have shown promise, N‐[2‐(1H‐indol‐3‐yl)ethyl]hexanamide and N‐[2‐(1H‐indol‐3‐yl)ethyl]benzamide; they are able to block synchronization caused by the addition of melatonin to parasite culture, while being unable to modulate cycle progression on their own. In addition, three other derivatives have shown promise as new structures for the development of toxic derivatives, with IC50 values on the order of 10 µM and the possibility to insert structural modifications capable of decreasing this value further.^56^

Using reactions designed based on the structure of melatonin and derived compounds, Luthra et al (2019) developed a new class of antimalarials based on arylalkanimino tryptamine derivatives. Several compounds with antimalarial activity have been found in the low micromolar/high nanomolar range capable of blocking the erythrocytic cycle at the trophozoite stage and melatonin‐induced growth synchronization in parasite populations. In addition, binding studies indicated that some of the compounds tested were still capable of interacting with the human melatonin receptor MT1.^57^

Archibald L. Svogie et al (2015) described a new class of indole‐derived antimalarials with promising activity, indolyl‐3‐ethanone‐α‐thioethers. Their screening highlighted two compounds with good antimalarial activity, 1‐(1H‐indol‐3‐yl)‐2‐[(4‐nitrophenyl)thio]ethanone (IC50, 240 nM) and 1‐(5‐chloro‐1H‐indol‐3‐yl)‐2‐[(4‐bromophenyl)thio]ethanone (IC50, 90 nM), both with no toxicity in HeLa cells.^58^ Several other indole derivatives were synthesized by Lunga et al (2018), resulting in two new candidates with antimalarial activity, 1‐(5‐chloro‐1H‐indol‐3‐yl)‐2‐[(4‐cyanophenyl)thio]ethanone and 1‐(5‐chloro‐1H‐indol‐3‐yl)‐2‐[(4‐nitrophenyl)thio]ethenone. These two compounds had effects comparable to chloroquine, with IC50s in the range of 30 nM. All of the compounds tested had no toxicity in HeLa cells or hemolytic activity.^59^

Inhibitors of several tRNA synthetases present in *Plasmodium* are promising drug targets and have been well explored for the development of alternative therapies.^60^, ^61^, ^62^, ^63^, ^64^, ^65^, ^66^ There are two compartmentalized tryptophanyl‐tRNA synthetases (TrpRS) in *P falciparum*: one cytoplasmic molecule that participates in eukaryotic tRNA binding, and one that is directed to the apicoplast that participates in bacterial tRNA synthesis. The presence of a bacterial TrpRS in *P falciparum* highlights a drug‐like target already used by antibiotics against both Gram‐positive and Gram‐negative bacteria, which is of low toxicity in humans. Indolmycin, an indole similar to tryptophan, used as an inhibitor of TrpRS in the apicoplast has an IC50 on the order of 1 micromolar when the parasite is exposed to the compound for 96 hours.^67^ The high efficacy only after 96 hours indicates a mechanism of "late death" that is typical of drugs that affect the apicoplast, and this is of great relevance for combined treatment with fast‐acting drugs, such as artemisinins.^68^, ^69^, ^70^, ^71^

## 4 CONCLUSIONS

The growing reports of multidrug‐resistant *Plasmodium* parasites and first‐line drug treatment failures put pressure on the scientific community to seek new drugs or alternative treatments to combat malaria. One option is the use of compounds capable of interfering with the duration and coordination of *Plasmodium* intraerythrocytic cycle. These compounds would disrupt the coordination of the cycle and consequently allow the host immune system to be more effective at combating infection. They do not necessarily need to be toxic to parasites, but they should potentiate the effect of antimalarials currently used to bypass resistance and result in more effective treatments, as was demonstrated with luzindole.^22^ Melatonin stands out as a host factor capable of interfering with the parasite cycle. Thus, indole compounds and melatonin derivatives have been explored as potential new antimalarial candidates.

The indole derivatives melatotosil and triptosil showed clear agonist and antagonist effects, respectively, to melatonin, and they are excellent starting points for the intelligent design of drugs capable of interfering with the coordination of the intraerythrocytic cycle. Several tested indole compounds have resulted in the inhibition of parasite growth in the nanomolar range, which is comparable with the antimalarials used today. Among them, triazine‐indole and indole alkaloids stand out.^53^, ^54^ In addition, there are also indole compounds capable of causing "late death" by affecting the synthesis of proteins in the apicoplast, which increases interest in their use in therapies combined with fast‐acting drugs, such as artemisinin.

Currently, there are many indole compound structures with some antimalarial activity, and the presence of various mechanisms of action and different targets shows that this class of compounds is promising for the development of new drugs. Whether with a toxic effect on the parasite or interference in the coordination of the cycle, these compounds are promising candidates to circumvent resistance to antimalarials.

## CONFLICT OF INTEREST

The authors declare no conflict of interest.

## AUTHOR CONTRIBUTIONS

PHSP and CRSG involved in conceptualization, draft preparation, writing, review, and editing; CRSG acquired funding. Both authors have read and agreed to the published version of the manuscript.
